# Disease severity in K/BxN serum transfer-induced arthritis is not affected by IL-33 deficiency

**DOI:** 10.1186/ar4143

**Published:** 2013-01-16

**Authors:** Praxedis Martin, Dominique Talabot-Ayer, Christian Alexander Seemayer, Solenne Vigne, Céline Lamacchia, Emiliana Rodriguez, Axel Finckh, Dirk E Smith, Cem Gabay, Gaby Palmer

**Affiliations:** 1 Division of Rheumatology, Department of Internal Medicine, University Hospital of Geneva, 26 avenue Beau-Séjour, 1211 Geneva 14, Switzerland; 2 Department of Pathology and Immunology, University of Geneva School of Medicine, 1 rue Michel-Servet, 1211 Geneva 4, Switzerland; 3 Novartis Pharma AG, Translational Medicine, NIBR, WSJ386.10.48, PO box, CH-4002, Basel, Switzerland; 4 Department of Inflammation Research, Amgen Inc., 1201 Amgen Court West, Seattle, WA 98119, USA

## Abstract

**Introduction:**

Interleukin (IL)-33 is a cytokine of the IL-1 family, which signals through the ST2 receptor. Previous work suggested implication of the IL-33/ST2 axis in the pathogenesis of human and mouse arthritis. Here, we directly investigated the role of endogenous IL-33 in K/BxN serum transfer-induced arthritis by using IL-33 knockout (KO) mice.

**Methods:**

Arthritis was induced by injection of complete K/BxN serum or purified IgG. Disease severity was monitored by clinical and histological scoring.

**Results:**

K/BxN serum transfer induced pronounced arthritis with similar incidence and severity in IL-33 KO and wild-type (WT) mice. In contrast, disease development was significantly reduced in ST2 KO mice. IL-33 expression in synovial tissue was comparable in arthritic WT and ST2 KO mice, and absent in IL-33 KO mice. Transfer of purified arthritogenic IgG instead of complete K/BxN serum also resulted in similar arthritis severity in IL-33 KO and WT mice, excluding a contribution of IL-33 contained in the serum of donor mice to explain this result. We investigated additional potential confounding factors, including purity of genetic background, but the mechanisms underlying reduced arthritis in ST2 KO mice remained unclear.

**Conclusions:**

The data obtained with IL-33 KO mice indicate that endogenous IL-33 is not required for the development of joint inflammation in K/BxN serum transfer-induced arthritis. On the contrary, arthritis severity was reduced in ST2 KO mice. This observation might relate to IL-33 independent effects of ST2, and/or reveal the existence of confounding variables affecting the severity of joint inflammation in these KO strains.

## Introduction

Interleukin (IL)-33 is the most recently discovered member of the IL-1 cytokine family (see [1] for review). IL-33, like IL-1α, is a dual-function protein, displaying both nuclear and extracellular effects. The latter are mediated by its binding to an IL-1 receptor family member called ST2 (*IL1RL1*). ST2 exists in two isoforms generated by alternative splicing [2]. The short isoform (sST2) acts as a soluble decoy receptor [3]. The long signaling ST2 receptor isoform (ST2l) is expressed in many hematopoietic cells, including a number of innate immune cells, which are involved in T helper 2 (Th2)-type response. Consistently, injection of recombinant IL-33 induces or amplifies type 2 immune effects in different mouse models [4-6]. In addition IL-33 acts on neutrophils, Th1 cells, natural killer cells, and mast cells, as well as on endothelial cells, to induce proinflammatory effects, so that a role for IL-33 in host defense and immunopathology independently of Th2 immunity has also been suggested [7-9]. IL-33 is constitutively expressed in stromal cells, including epithelial cells and specialized fibroblasts, as well as in endothelial cells in human, but only to a limited extent in mouse [10,11]. It has been proposed that, upon tissue damage, constitutively expressed IL-33 leaks from necrotic cells and acts as an alarmin to initiate or amplify immune responses [10,12].

Previous work suggested a functional role of the IL-33/ST2 axis in the pathogenesis of human and mouse arthritis. In human rheumatoid arthritis (RA), IL-33 levels in serum and synovial fluid are elevated [13-15] and strong IL-33 expression can be detected in endothelial cells and fibroblasts in human RA synovium [16,17]. In mouse models of experimental arthritis involving active immunization, such as collagen- and antigen-induced arthritis, the use of ST2 knockout (KO) mice, ST2 blockade or injection of sST2 led to decreased immune responses and severity of arthritis, while injection of recombinant IL-33 increased arthritis severity, suggesting a pathogenic role for IL-33, signaling through ST2, in these experimental models [18-21]. Two studies investigated involvement of IL-33 in the inflammatory effector phase of arthritis, as it can be studied in the K/BxN serum transfer-induced model [22,23]. The first study concluded to a pathogenic role of IL-33 based on reduced arthritis severity in ST2 KO mice and increased disease severity after injection of IL-33 [22]. The second study reported an opposite effect of IL-33 injection, which suppressed joint inflammation by enhancing the production of Th2 cytokines and upregulating the inhibitory Fc receptor FcγRIIB on macrophages [23].

In the present study, we directly investigated the role of endogenous IL-33 in K/BxN serum transfer-induced arthritis using IL-33 KO mice and compared the results to those obtained using ST2 KO mice. We confirm decreased severity of arthritis in ST2 KO, but not in IL-33 KO mice, suggesting that endogenous IL-33, although expressed in the synovium, is not required for the development of arthritis in this model.

## Materials and methods

### Mice

C57BL/6 mice were obtained from Janvier (Le Genest-St-Isle, France). IL-33 KO mice (B6.129Sv-Il33) were generated at Amgen Inc. (Thousand Oaks, CA, USA) by targeting of the *Il33 *gene in 129Sv ES cells resulting in the deletion of six out of the seven coding exons [24]. Genotyping was performed by a 3-primer PCR combining a common forward primer (5'-TGC TGA ATT TTA TTC TCC CCC C-3') with reverse primers specific for the wild-type (WT) (5'-GCC CGT CTT CAT GTT GAA ATA-3') or the KO (5'-GCT CAT TCC TCC CAC TCA TGA-3') allele. IL-33 KO mice were backcrossed to the C57BL/6 background for six generations by speed congenics and considered to be 100% congenic based on analysis of a 377 SNIP panel (Taconic, Hudson, NY, USA). ST2 KO C57BL/6 mice (Il1rl1^tm1Anjm^, [25]) were obtained from the MRC Laboratory of Molecular Biology (Cambridge, UK). For arthritis experiments, local colonies of WT, IL-33 KO and ST2 KO mice were established at the Centre Médical Universitaire in Geneva. KRN T cell receptor transgenic mice [26] were provided by the Institut de Génétique et de Biologie Moléculaire et Cellulaire (Strasbourg, France) and maintained on a C57BL/6 background (K/B). NOD/Lt mice were purchased from Charles River (L'Arbresle, France). Mice were housed under conventional or low barrier conditions. Institutional approval was obtained for all animal experiments (Geneva Veterinarian Office, licenses 31.1.1005/3402/2, 3402/2-C and 3402/2-R).

### Biological reagents

Blocking anti-ST2, anti-IL-1RI and isotype-matched control antibodies were generated at Amgen Inc. Cell culture media were obtained from Invitrogen Life Technologies (Basel, Switzerland). Recombinant mouse IL-33 was purchased from Enzo Life Sciences (Lausen, Switzerland), human IL-1β and mouse IL-18 from R&D Systems (Abingdon, UK), and purified lipopolysaccharide (LPS) from Fluka (Escherichia coli 055:B5, Buchs, Switzerland). Murine IL-36β was produced at Amgen Inc. as an N-terminal truncation variant [27].

### K/BxN serum transfer-induced arthritis

Arthritic K/BxN mice were generated by crossing K/B mice with NOD/Lt mice, adult arthritic K/BxN mice were bled, and the sera were pooled. Age-matched female recipient WT, IL-33 KO or ST2 KO C57BL/6 mice were injected with pooled serum (200 μl, i.p.) on days 0 and 2. Alternatively, WT C57BL/6 mice were injected with pooled serum (200 μl, i.p.) on days 0 and 2, as well as with a monoclonal murinized immunoglobulin G (IgG1)-blocking anti-ST2 antibody (150 μg/mouse), a monoclonal murinized IgG1-blocking anti-IL-1R1 antibody (150 μg/mouse), or a monoclonal mouse IgG1 antibody directed against an irrelevant human antigen that was used as an isoytpe-matched control antibody (150 μg/mouse), on day 0 (4 h before injection of K/BxN serum) and on day 3 (experiment 1), or on days 0 and 2 (4 h before each injection of K/BxN serum; experiment 2). Efficacy of the blocking anti-ST2 antibody was demonstrated previously [24,28]. The development of arthritis was assessed daily and the severity of arthritis was scored in a blinded fashion for each paw on a 3-point scale, in which 0 = normal appearance, 1 = localized edema/erythema over one surface of the paw, 2 = edema/erythema involving more than one surface of the paw, 3 = marked edema/erythema involving the whole paw. The scores of all four paws were added for a composite score. Mice were sacrificed on day 6.

### Purification of the total IgG fraction from K/BxN serum and induction of arthritis

Serum of arthritic K/BxN mice (20 ml) was purified on protein A/G Sepharose 4 Fast Flow (GE Healthcare Life Sciences, Glattbrugg, Switzerland) and dialyzed into PBS [29]. Recipient WT, IL-33 KO or ST2 KO mice were injected with the purified IgG fraction (450 μl i.p., corresponding to 200 μl of the initial volume of K/BxN serum) on days 0 and 2. Mice were assessed for arthritis severity daily and sacrificed on day 6.

### Histological grading of arthritis

At sacrifice, the right ankle joints were fixed in 10% buffered formalin, decalcified in 15% EDTA, and embedded in paraffin. Sections were stained with hematoxilin and eosin or toluidine blue and graded by one pathologist (CAS) in a blinded manner. The severity of the synovial inflammation including synovial hyperlasia and the % of polymorphonulcear (PMN) cell infiltration, as well as the degree of cartilage erosion and bone destruction were evaluated utilizing a scoring system ranging from 0 to 4 (0 = normal, 1 = minimal, 2 = moderate, 3 = severe, 4 = very severe) [30].

### RT-qPCR

Left ankles were harvested at sacrifice and RNA was extracted with Trizol (Invitrogen Life Technologies). Total RNA (1 μg) was treated with RQ1 DNAse (Promega, Madison, WI, USA) and reverse transcribed using SuperScript II Reverse transcriptase (Invitrogen Life Technologies). Cytokine mRNA levels were assessed by RT-qPCR using appropriate primers (Table 1) and iQ SYBR Green Supermix in the iCycler iQ™ Real-Time PCR Detection System (Bio-Rad Laboratories Inc., Hercules, CA, USA). The annealing temperature was 60°C. Non-reverse-transcribed RNA samples and water were included as negative controls. RNA expression levels were calculated using the comparative method (2^-ΔCt^) for relative quantification by normalization to *Gapdh *gene expression.

**Table 1:** Primer sequences for RT-qPCR analysis.

Target	GenBank	Primer	Primer sequence
Il33	NM_133775.2	Il33 fwd	5'-ggtgtggatgggaagaagctg-3'
		Il33 rev	5'-gaggactttttgtgaaggacg-3'
Il6	NM_031168.1	Il6 fwd	5'-tgaacaacgatgatgcacttgcaga-3'
		Il6 rev	5'-tctgtatctctctgaaggactctggct-3'
Il1r1	NM_008362.2	Il1r1 fwd	5'-gagttacccgaggtccagtgg-3'
		Il1r1 rev	5'-gagggctcaggataacagg-3'
Il1rl2	NM_133193.3	Il1rl2 fwd	5'-aaacacctagcaaaagcccag-3'
		Il1rl2 rev	5'-agactgcccgattttcctatg-3'
Il18r1	NM_008365.2	Il18r1 fwd	5'-ggagatgagggctactactcctgcg-3'
		Il18r1 rev	5'-gtcttcttgcacattagggtctgagctg-3'
Il18rap	NM_010553	Il18rap fwd	5'-cccggaagtgctagaagaca-3'
		Il18rap rev	5'-acccgcagagcctttttgac-3'
Gapdh	NM_008084.2	Gapdh fwd	5'-acggccgcatcttcttgtgca-3'
		Gapdh rev	5'-aatggcagccctggtgacca-3'

### Determination of cytokine levels in serum and tissue lysates

Serum and right wrist joints were harvested at sacrifice. Joints were homogenized in 500 μl of lysis buffer (50 mM Tris-HCl, pH 7.5, 150 mM NaCl, complete EDTA-free protease inhibitor cocktail (Roche Diagnostics AG, Rotkreuz, Switzerland)). Lysates were cleared by centrifugation and total protein content was determined with the DC protein assay kit (Bio-Rad Laboratories Inc.). IL-33 was quantified using a mouse IL-33 Milliplex cytokine magnetic beads assay (Millipore AG, Zug, Switzerland; detection limit 1.61 and 4.3 pg/ml in two different kits used). IL-6 levels were quantified using a DuoSet ELISA Development System (R&D Systems; detection limit 16 pg/ml).

### Immunohistochemistry

IL-33 protein expression was examined in knee joints of WT and ST2 KO mice using a polyclonal goat anti-IL-33 antibody (AF3626, R&D Systems). Formalin-fixed, decalcified, paraffin-embedded sections were deparaffinized and antigen retrieval was performed in a pressure chamber (Pascal; Dako, Baar, Switzerland) in 0.1 M Tris-HCl, pH 9.0, 0.01 M EDTA, at 125°C for 30 sec. Slides were blocked for endogenous peroxidase activity and incubated with anti-IL-33 antibody (1 μg/ml) in antibody diluent (number S2022, Dako) overnight at 4°C. Subsequently, slides were incubated with an anti-goat HRP antibody (1:500; number SC2304, Santa Cruz Biotechnology, Inc., Heidelberg, Germany) in antibody diluent and developed with diaminobenzidine (Dako). To assess staining specificity, negative controls were performed using knee sections of IL-33 KO mice.

### Cell culture

Bone marrow-derived dendritic cells (BMDC) were generated from WT, IL-33 KO and ST2 KO C57BL/6 mice as previously described [31]. On day 7, cells were seeded into triplicate wells of 96-well plates at 10^5 ^cells/ml and cultured without or with 100 ng/ml recombinant IL-33, IL-1β, IL-36β or IL-18 or LPS for 72 h. IL-6 production in culture supernatants was assessed by ELISA. Cells from triplicate wells were pooled and used for total RNA extraction using the RNeasy kit (Qiagen, Valencia, CA, USA). Expression of IL-1R family receptors was quantified by RT-qPCR using appropriate sets of primers (Table 1).

### Microsatellite marker genotyping

**Figure 1:**
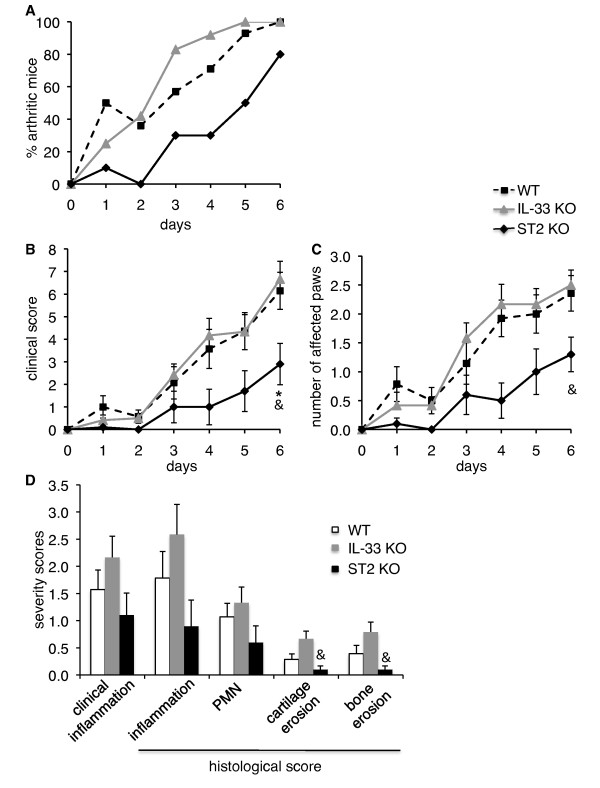
**Severity of serum transfer-induced arthritis is reduced in ST2, but not in IL-33 KO mice**. **(A) **Incidence of arthritis is shown for WT (*n *= 14, dashed line), IL-33 KO (*n *= 12, gray line), and ST2 KO (*n *= 10, black line) mice. Incidence was significantly retarded in ST2 KO, as compared to WT or IL-33 KO mice (*P *< 0.01, longitudinal model for binomial data). **(B-C) **Arthritis severity scores (B) and number of affected paws (C) are shown as the mean ± SEM for WT (dashed line), IL-33 KO (gray line), and ST2 KO (black line) mice. The evolution of severity scores (B; *P *= 0.001, mixed model for repeated measures) and final disease severity were significantly decreased in ST2 KO, as compared to WT (*P < 0.05) and IL-33 KO (&*P *< 0.05) mice. Evolution of the number of affected paws tended to be lower in ST2 KO, as compared to IL-33 KO and WT mice (*P *= 0.05, longitudinal model for ordinal data). **(D) **Clinical scores for inflammation of the right hind paw, which was processed for histological evaluation, and histological scores for inflammation, polymorphonulcear cell (PMN) infiltration, cartilage and bone erosion are shown as the mean ± SEM for WT (open columns), IL-33 KO (gray columns), and ST2 KO (black columns) mice. Cartilage and bone erosion were significantly reduced in ST2 KO, as compared to IL-33 KO mice; &*P *< 0.05. KO, knockout; WT, wild-type.

IL-33 KO, ST2 KO and WT mice (*n *= 3 per genotype) were randomly selected at the end of the experiment shown in Figure 1 and genotyped for 55 microsatellite markers as described [32]. In addition, all ST2 KO mice used in the arthritis experiments described herein were screened for D1Mit3, D1Mit211, D1Mit75a, D1Mit303, D6Mit166, D6Mit159, D6Mit102, and D15Mit193 genotypes using appropriate primers ([32]; see [33] for additional primer information).

### Statistical analysis

To assess differences in the longitudinal evolution of arthritis outcomes, we used generalized linear mixed models for repeated measures [34]. Tests were conducted at error alpha level of 0.05, two-sided and performed with STATA v. 11 for Windows (StataCorp LP, College Station, TX, USA). Differences in arthritis severity at the end of the follow-up were evaluated using the Kruskal-Wallis test. Significance of differences in histological scores, cytokine measurements, and mRNA expression was assessed by ANOVA or Kruskal-Wallis test, as appropriate.

## Results

### IL-33 and ST2 KO mice display different phenotypes in K/BxN serum transfer-induced arthritis

We examined incidence and severity of K/BxN serum transfer-induced arthritis in IL-33 KO, ST2 KO and WT C57BL/6 mice. Clinical scoring showed reduced incidence and severity of arthritis in ST2 KO mice as compared to WT controls (Figure 1A-C). In contrast, arthritis development and severity in IL-33 KO mice was similar to the disease observed in WT mice. Histological scoring of arthritic ankles on day 6 confirmed a trend toward reduced inflammation and PMN cell infiltration, as well as reduced cartilage and bone erosion in ST2 KO paws (Figure 1D).

**Figure 2:**
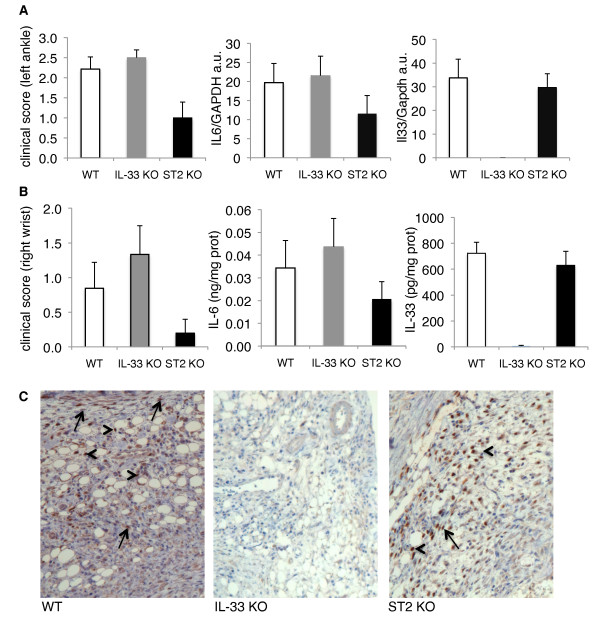
**Synovial expression of IL-33 during KBxN serum transfer-induced arthritis**. **(A) **Clinical scores for inflammation of the left hind paw, which was processed for RNA extraction are shown (left panel), along with expression levels for IL-6 (middle panel) and IL-33 (right panel) mRNAs in the joint. Transcript levels were normalized relative to Gapdh expression and represent the mean ± SEM for WT C57BL/6 (*n *= 14, open columns), IL-33 KO (*n *= 12, gray columns), and ST2 KO (*n *= 10, black columns) mice. **(B) **Clinical scores for inflammation of the right forepaw, which was processed for protein extraction are shown (left panel), along with IL-6 (middle panel) and IL-33 (right panel) protein expression levels in the joint. Cytokine production was normalized to total protein content and represents the mean ± SEM for WT C57BL/6 (*n *= 14, open columns), IL-33 KO (*n *= 12, gray columns), and ST2 KO (*n *= 10, black columns) mice. **(C) **IL-33 protein expression (nuclear brown staining) was assessed in the synovium of arthritic knee joints of WT (left panel), IL-33 KO (middle panel) and ST2 KO (right panel) mice by immunohistochemistry (IHC) (original magnifications x200). Nuclear IL-33 staining was observed in WT and ST2 KO mice in cells morphologically consistent with fibroblasts (arrows), as well as in other cell types, which were not formally identified (arrowheads). KO, knockout; WT, wild-type.

On day 6 after induction of arthritis, serum IL-33 levels were similar in WT and ST2 KO mice (WT, mean: 310 ± 192 pg/ml, range < 1.61 to 1258 pg/ml; ST2KO, 447 ± 287 pg/ml, range < 1.61 to 2142 pg/ml) and comparable to IL-33 levels measured in naïve WT C57BL/6 mice (*n *= 17; 294 ± 191 pg/ml, range < 1.61 to 3062 pg/ml) housed in our conventional mouse facility. IL-33 was undetectable in the serum of IL-33 KO mice. IL-33 mRNA (Figure 2A) and protein (Figure 2B) expression in synovial tissues was similar in arthritic WT and ST2 KO mice, and absent in IL-33 KO mice. Expression of IL-6 mRNA (Figure 2A) and protein (Figure 2B) in arthritic paws, monitored as a marker of inflammation, paralleled severity scores and tended to be lower in ST2 KO as compared to WT mice. Strong nuclear IL-33 immunostaining was observed in the synovial tissue of WT and ST2 KO mice, in cells morphologically consistent with fibroblasts, as well as in other cell types, which we have not formally identified (Figure 2C). Interestingly, and in contrast to human RA synovium [16,17] synovial endothelial cells did not express IL-33. A similar pattern of IL-33 protein expression was observed in the synovium of WT and ST2 KO mice, while no IL-33 staining was detected in IL-33 KO mice.

### IL-33 and ST2 KO mice display different phenotypes in arthritis induced by injection of arthritogenic IgG

**Figure 3:**
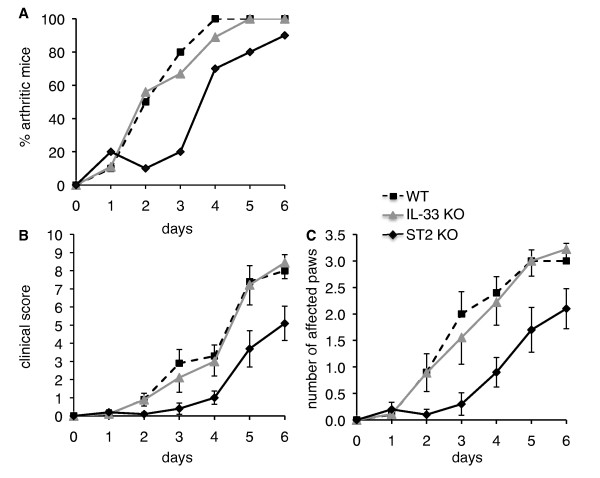
**IL-33 deficiency does not reduce severity of arthritis induced by IgG purified from K/BxN serum**. **(A) **Incidence of arthritis induced by total IgG purified from K/BxN serum is shown for WT C57BL/6 mice (*n *= 10, dashed line), IL-33 KO mice (*n *= 9, gray line), and ST2 KO mice (*n *= 10, black line). The incidence of arthritis was significantly retarded in ST2 KO, as compared to WT or IL-33 KO mice (*P *< 0.01, longitudinal model for binomial data). Arthritis severity was evaluated by clinical assessment of arthritis severity scores **(B) **and number of affected paws **(C) **in WT C57BL/6 mice (*n *= 10, dashed line), IL-33 KO mice (*n *= 9, gray line), and ST2 KO mice (*n *= 10, black line). Results shown represent the mean ± SEM for each group of mice. The evolution of severity scores (B; *P *< 0.01, mixed model for repeated measures) and of the number of affected paws (C, *P *< 0.001, longitudinal model for ordinal data) was significantly decreased in ST2 KO, as compared to IL-33 KO and WT mice. In contrast, incidence and severity of arthritis were similar in IL-33 KO and WT mice. IgG, immunoglobulin G; KO, knockout; WT, wild-type.

We wondered whether differences observed between IL-33 and ST2 KO mice regarding the severity of arthritis might be related to the existence of confounding variables in our experiment. Among these, we wanted to exclude a potential effect of IL-33 present in the injected serum, which might affect arthritis severity in IL-33-deficient, but not in ST2 receptor-deficient mice. The amount of IL-33 detected in different KBxN serum pools (*n *= 4) used for arthritis induction was 77 ± 31 pg/ml (range < 4.3 to 139 pg/ml). Therefore, to avoid co-injection of 'contaminating' IL-33 contained in the serum as a potential confounding factor, we thus used total IgG purified from K/BxN serum [29] to induce arthritis. IL-33 levels were below the detection limit of the Milliplex assay (4.3 pg/ml) in this purified IgG fraction. Similarly to the experiment performed with complete K/BxN serum, clinical scoring showed significantly reduced incidence and severity of arthritis in ST2 KO mice as compared to WT controls after injection of purified IgG (Figure 3A-C). In contrast, arthritis development and severity in IL-33 KO mice was still similar to that observed in WT controls. Therefore, a contribution of IL-33 contained in the serum of K/BxN donor mice to arthritis severity in IL-33 KO recipient mice can be excluded.

### Inhibition of ST2 signaling with a blocking antibody does not reduce K/BxN serum transfer-induced arthritis

**Figure 4:**
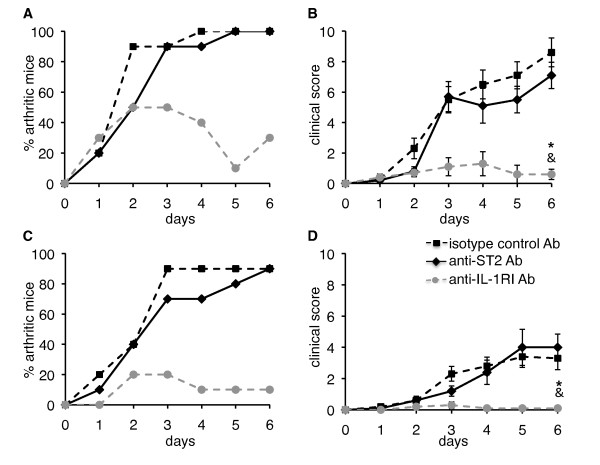
**Inhibition of ST2, respectively IL-1R signaling with blocking antibody**. **(A, C) **Incidence and **(B, D) **severity of K/BxN serum transfer-induced arthritis are shown for WT C57BL/6 mice treated with blocking anti-ST2 antibody (*n *= 10, black line), blocking anti-IL-1R1 antibody (*n *= 10, gray dashed line) or isotype control antibody (*n *= 10, black dashed line) in two independent experiments (A, B: experiment 1; C, D: experiment 2). Incidence of arthritis was markedly reduced (A, *P *< 0.01; C, *P *< 0.001, longitudinal model for binomial data) in anti-IL-1R1-treated, as compared to anti-ST2 or isotype control antibody-treated mice. (B, D) Arthritis severity scores are shown as the mean ± SEM for each group of mice. The evolution of arthritis severity scores (B, *P *< 0.001; D, *P *< 0.001, mixed model for repeated measures) and disease severity at the end of the experiment were significantly reduced in anti-IL-1R1 antibody-treated, as compared to anti-ST2 or isotype control antibody-treated mice (*P < 0.01 anti-IL-1R1 vs. isotype-control; &*P *< 0.01 anti-IL-1R1 vs. anti-ST2). Incidence and severity of arthritis were similar in anti-ST2 and isotype control antibody-treated mice in both experiments. WT, wild-type.

In an attempt to determine whether reduced arthritis severity in ST2 KO mice might relate to a true IL-33 independent effect of ST2 rather than to the existence of confounding variables unrelated to ST2, we used a blocking anti-ST2 antibody [24,28] to inhibit ST2 signaling during K/BxN serum transfer-induced arthritis (Figure 4). Treatment of the mice with the anti-ST2 antibody before and during development of arthritis did not reduce disease severity in two independent experiments, while blocking of the type 1 IL-1R with an isotype-matched anti-IL-1R1 blocking antibody essentially abrogated disease (Figure 4A-D). Histological severity scores confirmed clinical severity data (data not shown).

### Expression and function of other IL-1R family members in IL-33 and ST2 KO mice

**Figure 5:**
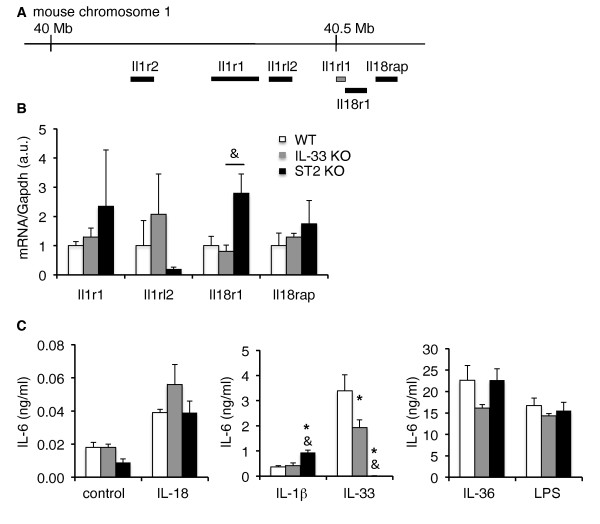
**Expression and function of other IL-1R family members in IL-33 KO and ST2 KO BMDC**. **(A) **Schematic representation of the IL-1R gene cluster, including the ST2 gene *Ilrl1*, on mouse chromosome 1. Mb, megabase pairs. **(B) **Expression levels of mRNA for different IL-1R family members encoded in this locus in WT, IL-33 KO and ST2 KO BMDC. Transcript levels were normalized to Gapdh expression and expressed relative to values obtained in WT cells, which were arbitrarily set to 1. Data shown represent the mean ± SEM of three determinations in independent cultures derived from IL-33 KO mice (grey columns), ST2 KO mice (black columns) or WT C57BL/6 mice (open columns). &*P *< 0.05 ST2KO vs. IL-33 KO cells, ANOVA. **(C) **Function of different IL-1R family members in cultured WT, IL-33 KO and ST2 KO BMDC. Induction of IL-6 production in response to 72 h of stimulation with different IL-1 family cytokines, namely IL-36, IL-1β, IL-18 and IL-33 (100 ng/ml) was monitored in BMDC derived from IL-33 KO mice (grey columns), ST2 KO mice (black columns) or WT C57BL/6 mice (open columns). Lipopolysaccharide (LPS) (100 ng/ml) stimulation was used as an unrelated positive control. Results are shown as mean ± SEM of values obtained in three independent cultures. *P < 0.05 vs. WT cells; &*P *< 0.05 ST2KO vs. IL-33 KO cells, ANOVA. BMDC, bone marrow-derived dendritic cell; KO, knockout; WT, wild-type.

We next wondered whether the decreased severity of arthritis observed in ST2 KO mice might be related to off-target effects of the KO construct, rather than to deletion of ST2 itself. The ST2 KO mice used in this study were generated by replacement of exons 4 and 5 of the ST2 gene with a neomycin selection cassette, which was left in the locus [25]. There are many examples where selection markers remain within targeted loci interfere with the expression of neighboring genes or even entire loci [35,36], so that nowadays more refined KO strategies, allowing for the subsequent removal of selection markers, are generally preferred. The mouse *Il1rl1 *gene encoding the ST2 receptor is located on chromosome 1, within a cluster of genes encoding other receptors of the IL-1R family, namely the type II decoy receptor for IL-1 (*Il1r2*), the type I signaling IL-1R (*Il1r1*), the receptor for IL-36 (IL-36R, *Il1rl2*), as well as the receptor and co-receptor for IL-18 (IL-18R, *Il18r1 *and IL-18R accessory protein, *Il18rap*) (Figure 5A). Interference of ST2 targeting with expression of any of these genes might in principle affect arthritis severity. IL-1 activity, in particular, is known to be absolutely required for joint inflammation in this model [37]. We thus examined expression of the signaling IL-1, IL-36 and IL-18 receptors (Figure 5B) and the responsiveness to cognate IL-1 family cytokines (Figure 5C) *in vitro *in cultured BMDC derived from ST2 KO mice, as compared to BMDC derived from WT and IL-33 KO mice. BMDC were chosen as target cells because they respond to all IL-1 family cytokines [31]. No significant differences in IL-1, IL-36 and IL-18 receptor expression were observed in BMDC derived from IL-33 KO or ST2 KO mice, as compared to WT cells (Figure 5B). Furthermore, the response of IL-33 KO and ST2 KO cells to IL-36 and IL-18, as assessed by induction of IL-6 production, was comparable to that of WT cells, while the response to IL-1β was even slightly enhanced in ST2 KO cells. The response to IL-33 was of course lost in ST2 KO cells and appeared to be also slightly reduced in IL-33 KO cells (Figure 5C). LPS, used as an unrelated positive control, elicited similar IL-6 production in IL-33 KO, ST2 KO and WT BMDC. These data do not point toward any major defect in expression of other IL-1R family receptors or in IL-1 family cytokine signaling in ST2 KO cells.

### Influence of genetic background on arthritis severity in ST2 KO mice

**Figure 6:**
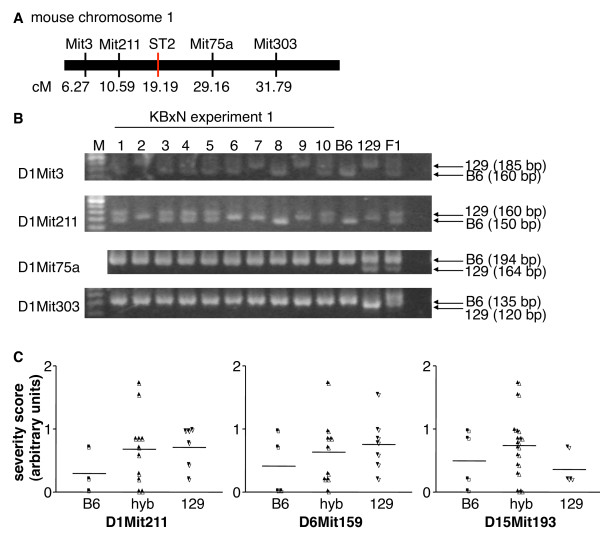
**Influence of 129 genetic background carryover on arthritis severity in ST2 KO mice**. **(A) **Schematic representation of markers screened on chromosome 1, cM 0-32. **(B) **Heterogeneity of genetic background in ST2 KO mice upstream of the ST2 locus on chromosome 1, as assessed by size differences in markers D1Mit3 and D1Mit211, in the ST2 KO (*n *= 10, lanes 1 to 10) mice used for the arthritis experiment shown in Figure 1. M, size ladder; B6, C57BL/6 control DNA; 129, 129SvPas control DNA; F1, B6x129F1 hybrid control DNA; bp, base pairs. **(C) **Correlation of D1Mit211 (left panel), D6Mit159 (middle panel) and D15Mit193 (right panel) genotypes (B6, hybrid or 129) with maximal clinical arthritis severity scores in the ST2 KO mice (*n *= 20) used for the arthritis experiments shown in Figure 1 and 3. Absolute values for arthritis severity scores were corrected for overall mean maximal severity in each experiment. KO, knockout.

IL-33 KO and ST2 KO mice were generated in 129Sv, respectively 129P2/OlaHsd ES cells and backcrossed to a C57BL/6 background. It has become increasingly clear that despite extensive backcrossing, genetic material of the original mouse strain, and in particular regions closely linked to the targeted loci, remains associated to KOs in recipient strains, sometimes leading to confounding effects mistakenly attributed to the disrupted gene [38,39]. We thus wondered whether carryover of 129 genetic material might have influenced the outcome of our experiments. IL-33 KO mice were considered 100% C57BL/6 congenic at the end of their backcross based on screening of a 377-marker panel. In addition, at the end of the experiment shown in Figure 1, we screened three mice per genotype using a 55-marker panel [32] to obtain a picture of the genetic background in our local mouse colonies. We confirmed 98% purity of the C57BL/6 background in WT (one heterogeneous marker: D4Mit166) and 100% purity in IL-33 KO mice. Screening of ST2 KO mice revealed lower (89%) purity (six heterogeneous markers: D1Mit211, D6Mi166, D6Mit159, D6Mit102, D11Mit224, D15Mit193). We further examined two additional markers close to the *Il1rl1 *gene on chromosome 1 (D1Mit3 and D1Mit75a, Figure 6A, Table 2), one of which (D1Mit3) was also non-C57BL/6 in 17 out of 20 ST2 KO mice tested (Figure 6B and data not shown). In contrast, the D1Mit75a and D1Mit303 markers, located downstream of the ST2 locus were C57BL/6 in all ST2 KO mice examined. Finally, we screened the 20 ST2 KO mice used in arthritis experiments (Figure 1 and 3) for the presence of either C57BL/6, or 129 alleles at the D1Mit211, D6Mi166, D6Mit159, D6Mit102, and D15Mit193 loci and correlated their genotypes with arthritis severity of individual mice (Figure 6C and data not shown). With the limited number of mice included in these studies, we observed no significant correlations. However, arthritis severity in mice carrying two C57BL/6 alleles at a given locus often tended to be lowest (D1Mit211, D6Mit166; D6Mit159).

**Table 2:** Primer sequences for chromosome 1 microsatellite analysis.

Target	Primer name	Primer sequence
D1Mit3	D1Mit3 fwd	5'-tttttgttttcttttcttttccc-3'
	D1Mit3 rev	5'-ccctcttctggtttccacat-3'
D1Mit211	D1Mit211 fwd	5'-gttattcatcaaaatacagatggcc-3'
	D1Mit211 rev	5'-tctgctgctaagtagaatgaatgc-3'
D1Mit75a	D1Mit75a fwd	5'-aaatcaccatggatttggttg-3'
	D1Mit75a rev	5'-cctgtgccaacaaattaccc-3'
D1Mit303	D1Mit303 fwd	5'-ggtttctatttcggttctcgg-3'
	D1Mit303 rev	5'-tctgtgctgcaaaacagagg-3'

## Discussion

Our results indicate that, although IL-33 is expressed in the synovium during K/BxN serum transfer-induced arthritis, IL-33 KO mice display similar arthritis severity as compared to WT controls, suggesting that endogenous IL-33 is not required for the development of joint inflammation in this experimental model. Contrastingly, disease severity was reduced in ST2 KO mice.

We thus confirm previous data indicating that ST2-deficient mice have reduced severity of serum transfer-induced arthritis [22]. However, this effect seems unrelated to the role of endogenous IL-33 and we have not been able to determine precisely why ST2 KO display less severe arthritis. The reduction of joint inflammation in ST2 KO mice is consistent in our hands and in experiments performed by others [22] and this observation certainly merits further investigation. Interestingly, we observed not only reduced inflammation, but also reduced bone erosion in ST2 KO mice. This finding is different from data reported previously [40,41], describing a deleterious effect of the ST2 KO on bone, at steady state and in the context of TNF-α-induced arthritis. In contrast, in the present highly inflammatory model of arthritis, bone loss appeared to correlate mostly with the degree of inflammation, rather than being associated with a particular genotype.

In the course of these experiments, we observed nuclear expression of the IL-33 protein in the arthritic synovium. However, in striking contrast with human synovium [17] and consistent with data in other mouse tissues [11], IL-33 expression was not detected in endothelial cells in arthritic mouse paws. This observation emphasizes important differences in IL-33 biology between human and mouse, which need to be taken into account when inferring IL-33 function in human physiology or pathology from mouse data.

Discrepant observations in studies using different ST2 KO lines, or even in different studies using the same mouse line, as well as between ST2 KO and IL-33 KO mice, have already been reported in other models, and in particular in the context of allergic airway inflammation [42-45]. However, this study is, to our knowledge, the first instance where IL-33 and ST2 KO mice are compared side by side in the same experiment. Our observation that endogenous IL-33 is not required for the development of joint inflammation in serum transfer-induced arthritis differs from previous conclusions based on indirect evidence obtained using ST2 KO mice and injections of recombinant IL-33 [22,23], suggesting that caution is warranted when extrapolating such data to conclude on functions of endogenous IL-33, as already highlighted by other studies [46-48].

We considered a number of potential confounding variables to explain our discrepant observations in IL-33 and ST2 KO mice. Among these, we excluded contamination of injected serum with IL-33 to explain the lack of difference between IL-33 KO and WT mice. We also analyzed the potential interference of ST2 gene targeting with expression of other IL-1R family receptors encoded in the same gene cluster on mouse chromosome 1. Using BMDC isolated from WT, IL-33 KO or ST2 KO mice, we found no evidence for a major defect in expression or function of one of these receptors in ST2 KO cells. However, based on our limited set of data, we cannot exclude more subtle alterations in gene expression or signaling that might affect arthritis severity. Furthermore, we serendipitously observed that, although ST2 KO cells lack surface expression of ST2l ([25] and our unpublished observations), and display no functional response to IL-33 [49], the 3' part of the ST2l mRNA is still expressed in ST2 KO cells and tissues, to levels similar to those detected in WT mice (PM *et al.*, unpublished observations). By 5'RACE, we localized the 5' end of the truncated ST2l transcript in ST2 KO cells to exon 8 of the ST2 gene (bp 1081 of the ST2l cDNA, GenBank accession NM_001025602). This transcript includes an open reading frame potentially encoding a truncated ST2l protein starting at amino acid 346, which corresponds to the beginning of the intracellular domain of the receptor. Although, due to lack of a suitable antibody recognizing the intracellular part of mouse ST2l, we have not been able to verify expression of a truncated protein in ST2 KO mice, expression of a free ST2 Toll/IL-1R (TIR) domain might conceivably interfere with signaling of other IL-1R family receptors, by analogy to regulation of IL-1R and Toll-like receptor (TLR) signaling by the IL-1R family member SIGIRR [50]. Interestingly, and whatever the mechanism involved, there is previous evidence for complex alterations in the response to another IL-1 cytokine in ST2 KO cells, with basophils of ST2 KO mice showing decreased responses to IL-18 in terms of IL-4 and IL-13, but not IL-6, production, as compared to WT cells [7].

We also considered the potential existence of microRNAs (miRNAs) within and around the ST2 locus, the expression of which might have been affected by ST2 targeting. To date, by searching miRBase [51], we found no miRNA described in proximity of the *Il1rl1 *gene (chromosome 1; 40.5 Mb), the closest miRNAs reported being mmu-mir-5103 (miRBase accession number MI0018011; 34.5 Mb) and mmu-mir-1928 (MI0009917; 74.3 Mb).

A previous study described higher severity of K/BxN serum transfer-induced arthritis in C57BL/6, as compared to 129 WT mice [52], and we wondered whether carryover of genetic material from the original 129 strains might have affected arthritis severity in the IL-33 and ST2 lines used in this study. While IL-33 KO mice were backcrossed to high purity to C57BL/6, the backcross was less complete in ST2 KO mice, and we observed persistence of non-C57BL/6 markers on chromosome 1, close to the ST2 locus, but also on chromosomes 6, 11 and 15. However, in the regions examined, arthritis severity often tended to be even less severe in the few mice, which carried two C57BL/6 alleles, so that we cannot provide evidence for any obvious effect of 129 carryover in decreasing joint inflammation. Nevertheless, more complex genetic interactions among multiple chromosomal sites acting to affect disease severity in these mice cannot be excluded based on this limited analysis.

Obviously, an alternative explanation for the difference in arthritis severity observed between ST2 and IL-33 KO mice might lie with the existence of another ligand for ST2, although we are not aware of data designating a candidate molecule for this function and we have been unable to demonstrate any substantial binding interaction between ST2 and any other IL-1 family member besides IL-33. In an attempt to determine whether reduced arthritis severity in ST2 KO mice might relate to a true IL-33 independent effect of ST2, we used blocking anti-ST2 antibodies to inhibit ST2 signaling during K/BxN serum transfer-induced arthritis. In these experiments, treatment of the mice with anti-ST2 antibodies did not reduce disease severity, while blocking of the type 1 IL-1R with an isotype-matched anti-IL-1R1 blocking antibody essentially abrogated disease. This observation suggests a minor contribution, if any, of ST2 signaling to disease severity, as opposed to a major contribution of IL-1R1, consistent with the previously reported critical role of IL-1 in arthritis pathogenesis in this model [37]. The anti-ST2 antibody used efficiently prevented the biological activity of recombinant IL-33 *in vitro *and injected *in vivo *[24,28]. However, it is unknown whether the IL-33 blocking ability of this antibody would translate into the blocking of an IL-33-independent activity. Furthermore, although suggested by the efficacy of the anti-IL-1R1 antibody, complete target coverage of the antibody within the joint over the whole duration of the arthritis experiment seems difficult to verify. Therefore, although we consider it unlikely, we cannot formally exclude a marginal role of ST2 signaling in the pathogenesis of K/BxN serum transfer-induced arthritis.

In contrast to our present observations, we previously reported that treatment with a blocking anti-ST2 antibody efficiently reduced disease severity in collagen-induced arthritis, [18-21]. In fact, in mouse models of arthritis involving active immunization, such as collagen- or antigen-induced arthritis, the use of ST2 KO mice, injection of anti-ST2 antibodies or treatment with sST2 consistently led to decreased immune responses and severity of arthritis [18-21]. Conversely, injection of recombinant IL-33 enhanced disease severity, suggesting a role for IL-33, signaling through ST2, in modulating the pathogenic adaptive immune responses in these models. Nevertheless, in the light of our present work, it needs to be stressed that the contribution of endogenous IL-33 has not been directly investigated in this context and remains to be formally demonstrated.

## Conclusions

These data indicate that, while IL-33 is expressed in the synovium during K/BxN serum transfer-induced arthritis, IL-33 KO mice displayed similar arthritis development and severity as WT controls. Contrastingly, disease severity was reduced in ST2 KO mice; however, genetic analysis revealed that the degree of backcrossing in ST2 KO mice is much less complete. It is unclear, therefore, whether the difference between IL-33 and ST2 KO mice relates to IL-33 independent effects of ST2 or, instead, the existence of confounding variables affecting the severity of joint inflammation in these KO strains.

## Abbreviations

BMDC: bone marrow-derived dendritic cell; bp: base pair; ELISA: enzyme-linked immunosorbent assay; IgG: immunoglobulin G; IL: interleukin; KO: knockout; LPS: lipopolysaccharide; miRNA: microRNA; PBS: phosphate-buffered saline; PMN: polymorphonulcear cell; qRT-PCR: quantitative reverse transcription-polymerase chain reaction; RA: rheumatoid arthritis; TIR: Toll/interleukin-1 receptor; TLR: Toll-like receptor; TNF-α: tumor necrosis factor alpha; WT: wild-type.

## Competing interests

CAS is en employee of Novartis Pharma AG, Basel, Switzerland. DES is an employee of Amgen Inc., Seattle, WA, USA. The other authors declare they have no competing interests.

## Authors' contributions

PM planned the studies, performed experiments, analyzed data and wrote the manuscript. DTA, SV, CL and ER did experiments and analyzed data. CAS performed the histological scoring, analyzed data and wrote the manuscript. AF analyzed data and wrote the manuscript. DES generated the knockout mice, analyzed data and wrote the manuscript. CG planned the studies, analyzed data and wrote the manuscript. GP supervised the project, planned the studies, performed experiments, analyzed data and wrote the manuscript. All authors read and approved the final manuscript.
